# Development of DNA markers using next-generation sequencing approach for molecular authentication of *Boerhavia diffusa* L. and *Tinospora cordifolia* (Willd.) Miers

**DOI:** 10.1007/s13205-023-03732-7

**Published:** 2023-08-15

**Authors:** Anu Radha Sharma, Manik Vohra, Chigateri M. Vinay, Bobby Paul, Sanjiban Chakrabarty, Padmalatha S. Rai

**Affiliations:** 1 Department of Biotechnology, Manipal School of Life Sciences, Manipal Academy of Higher Education, Manipal, 576104 India; 2 Department of Bioinformatics, Manipal School of Life Sciences, Manipal Academy of Higher Education, Manipal, India; 3 Department of Cell and Molecular Biology, Manipal School of Life Sciences, Manipal Academy of Higher Education, Manipal, India

**Keywords:** Next-generation sequencing, Suppressive subtractive hybridization, *Boerhavia diffusa* L., *Tinospora cordifolia* (Willd.) Miers, DNA markers

## Abstract

The adulteration of plants and their materials used in herbal formulations poses a severe health concern. Hence, there is a need to establish a reliable, cost-effective, and robust molecular biomarker to distinguish among species and identify herbal plants and raw drugs from adulterants. The present study used suppressive subtractive hybridization and next-generation sequencing technology to identify novel DNA markers for *Boerhavia diffusa* L. and *Tinospora cordifolia* (Willd.) Miers*.* We identified two primer sets for *B. diffusa *and one for *T. cordifolia.* The DNA markers were validated in different accessions of *B. diffusa* and *T. cordifolia and their* common adulterants to determine the sensitivity and specificity of developed DNA markers. The designed DNA markers showed 100% sensitivity and specificity in detecting *B. diffusa* and *T. cordifolia* from their adulterants. The strategy described here can be extrapolated for developing DNA markers to authenticate other plant species.

**Supplementary Information:**

The online version contains supplementary material available at 10.1007/s13205-023-03732-7.

## Introduction

The existence of herbal medicines in many discrete cultures dates from the ancient past. Around 250,000 different plant species are globally known to exist (Christenhusz and Byng 2016). Pharmaceutical and herbal medicines primarily drive the international market. With the growing demand in the world, there is a massive opportunity to export to the Indian herbal industry (Christenhusz and Byng 2016). However, the major hurdle is the global adulteration issue in the case of herbal medicines. It has been observed that the accidental substitution of plant materials is another growing issue that needs to be addressed by the pharmaceutical industry for the efficacy and safety of the beneficiaries of medicinal plant materials. This mainly occurs due to the same vernacular names for biologically distinct plants (Mitra and Kannan 2007). In addition, identifying plants using traditional methods is sometimes difficult, especially in cases where the material is derivative of a processed part of a plant or is present in powdered form.

With the growing issue of adulteration, establishing a stable universal molecular marker for identifying the source of the species has become of utmost necessity. There is a bend in molecular techniques to generate universal standards for DNA barcoding (Barcaccia et al. 2016; Singh 2018). The barcodes are used to check the universality, quality, sequence coverage, and discrimination among different species. The existing DNA barcodes for terrestrial plants include *rbcL, matK, ITS, trnH-psbA*, etc. (Taberlet et al. 2007; Vijayan and Tsou 2010; Cabelin and Alejandro 2016). The methods used for genomic authentication, however, include restriction fragment length polymorphism (RFLP), random amplification of polymorphic DNA (RAPD), simple sequence repeats (SSR), and amplified fragment length polymorphism (AFLP). Still, none of them assure 100% authenticity (Semagn et al. 2006). Moreover, with the advancements in sequencing technologies, investigations are being conducted to evaluate the potential of semiconductor-based platforms for genotyping by sequencing various samples (Mascher et al. 2013). Hence, there is a need to establish a reliable, robust, cost-effective molecular biomarker to distinguish among species and identify herbal plants from adulterants of the same vernacular names.

The plants, namely, *Boerhavia diffusa* L. and *Trianthema portulacastrum* L. are addressed by the common vernacular Punarnava, and *Tinospora cordifolia* (Willd.) Miers and *Tinospora sinensis* (Lour.) Merr. are both addressed by the common name Guduchi. Traditionally Punarnava is used to treat diabetes, inflammation, cancer, prostatic hyperplasia, and gastrointestinal problems (Mishra et al. 2014). The bioactive compounds in *B. diffusa* include tannins, alkaloids, flavonoids, steroids, glycosides, terpenoids, rotenoids, and phenolic compounds (Parmar et al. 2018; Kaur 2019). The formulations from such combinations are used to treat various human ailments such as sciatica, inflammation, heart disease, spleen disease, abdomen, liver, and arthritis (Mishra et al. 2014). *T. cordifolia* on the other hand, contains terpenoids, steroids, alkaloids, lignans, and other compounds that bring about the plant's pharmacological activity (Sharma et al. 2019). In the traditional Indian system of medicine (Ayurveda), *T. cordifolia* has various therapeutic properties and is used to treat rheumatism, jaundice, skin disease, urinary disorder, diabetes, inflammation, anemia, allergic conditions, etc. (Goel et al. 2004; Sonkamble 2015). The roots of the *T. cordifolia* are used for bowel obstruction and are potent emetic.

To address the issue of adulteration with related plant species or common adulterants, this study was undertaken to identify novel DNA markers for *B. diffusa* and *T. cordifolia* utilizing suppressive subtractive hybridization coupled with next-generation sequencing (NGS) technology*.* This strategy can further be used to generate DNA markers to authenticate other plant species of medicinal and economic importance.

## Materials and methods

### Sample collection and identification

**Fig. 1:**
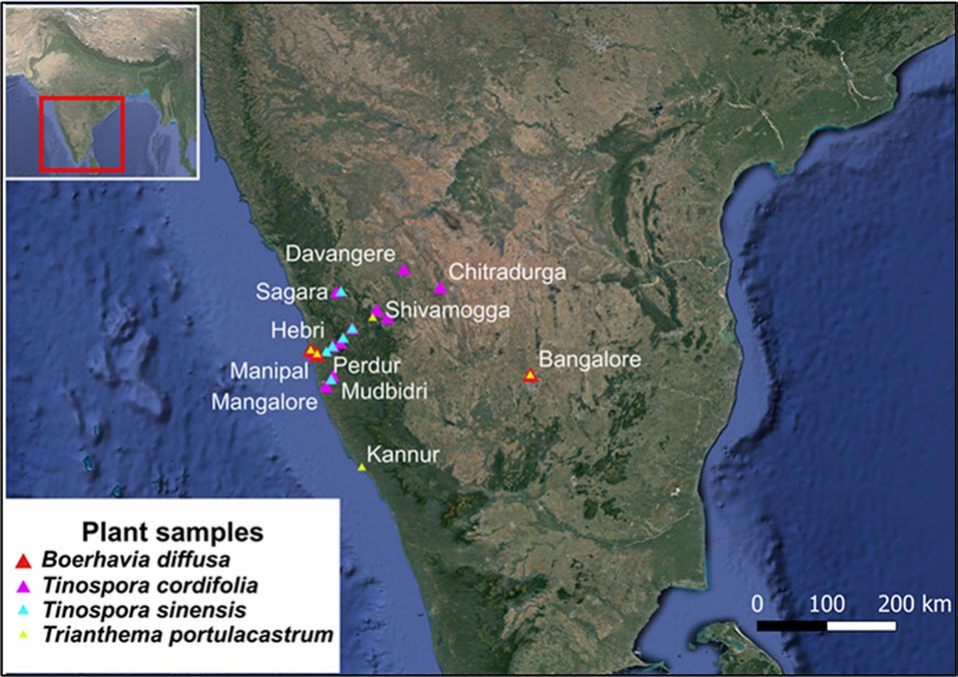
Geographical mapping of sample collection sites in Southern India

The two medicinal plants selected for the present investigation were *B. diffusa* (Punarnava) and *T. cordifolia* (Guduchi), belonging to the Ayurvedic Pharmacopoeia of India. These two plants form the main constituents of the Punarnava churna and Guduchi churna, that are used in ayurvedic treatments. Two other plants, namely *T. portulacastrum* (Punarnava) and *T. sinensis* (Guduchi) have similar vernacular names and are commonly used as adulterants or substitutes for Punarnava and Guduchi churna. The samples for selected plants were collected from Southern India (Fig. 1). Each plant species was assigned unique accession numbers, and a herbarium was prepared. The herbarium was used for taxonomical identification and was preserved for future reference. Germplasm of the selected plant species was maintained in the greenhouse at Manipal School of Life Sciences, MAHE, Manipal, India.

### Nucleic acid isolation and DNA barcoding

The DNA was isolated from the fresh and dried tissues of the collected plants and churna powder using a modified CTAB extraction method (Tiwari et al. 2012). The isolated DNA was checked for quality and quantity using 0.8% agarose gel. The samples were stored at – 80 °C until used for analysis. The DNA isolated from taxonomically identified fresh samples was subjected to molecular authentication using universal markers, i.e., *nrITS* and *matK*, by Sanger DNA sequencing. In brief, PCR was performed using universal primers. For *nrITS*, forward 5´-TCCTCCGCTTATTGATATGC-3´ and reverse 5´-CCTTATCATTTAGAGGAAGGA-3´ (Kress et al. 2005) primers, and for *matK*, forward 5ˈ—GTTCTAGCACAAGAAAGTCGA—3ˈ and reverse 5´-CTCAGATTATGATATTATTGA-3´ (Kress and Erickson 2008) primers were used. The PCR amplification was carried out in a thermal cycler (Applied Biosystems, USA) with the following conditions for the *nrITS* marker—initial denaturation step at 95 °C for 10 min; followed by 30 cycles at 95 °C for 1 min, 52 °C for 30 s and 72 °C for 1 min 30 s; and final extension period at 72 °C for 10 min. Similarly, for the *matK* marker, the PCR amplification conditions were the initial denaturation step at 95 °C for 10 min; followed by 30 cycles at 95 °C for 1 min, 48 °C for 30 s and 72 °C for 1 min 30 s; and final extension period at 72 °C for 10 min. The PCR products were purified using the magnetic beads purification method (MagBio Genomics, Gaithersburg, MD, USA) and subjected to sequencing on ABI 3130 sequencer.

### Suppressive subtractive hybridization and next-generation sequencing

**Fig. 2:**
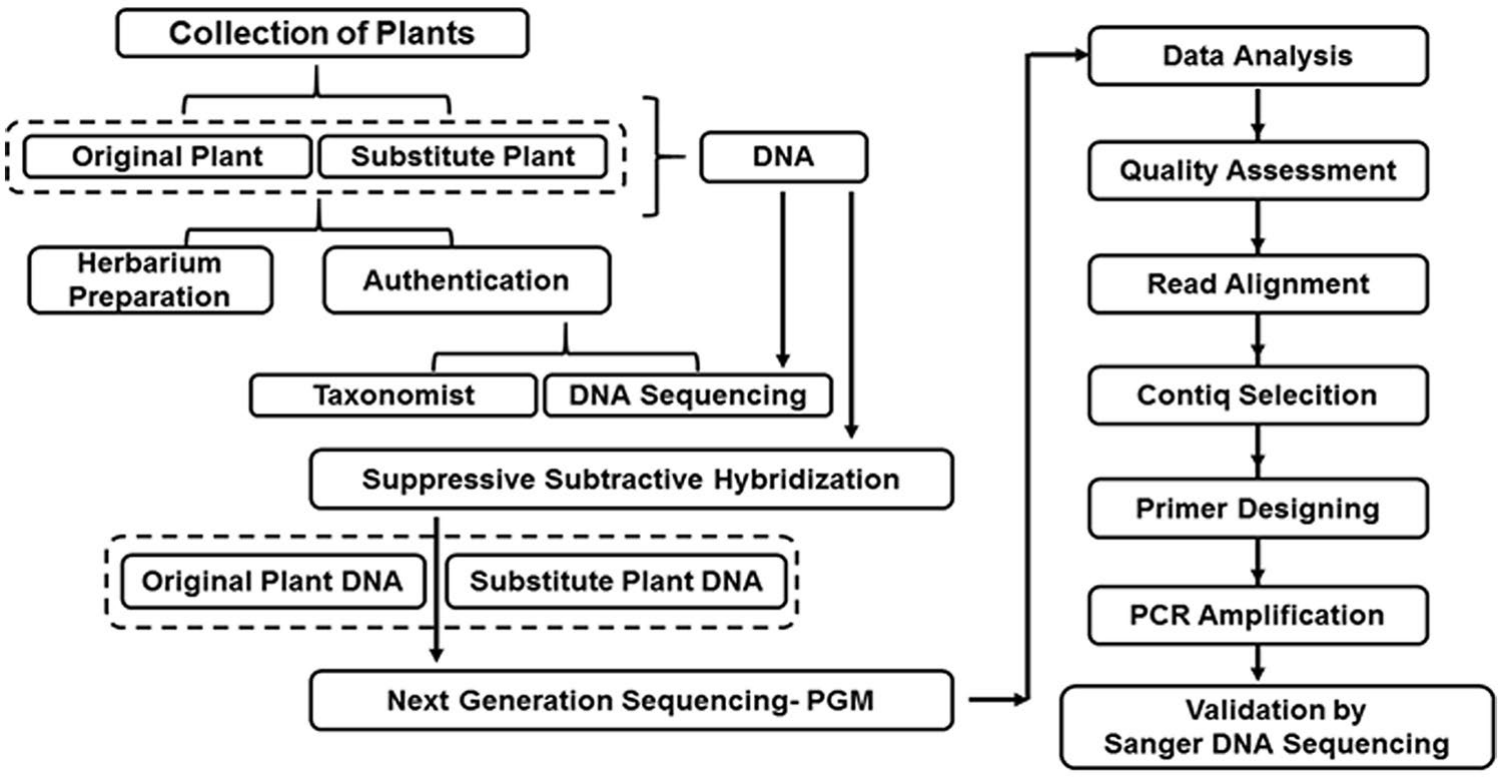
Strategy for the generation of DNA marker

We developed a strategy by coupling suppressive subtractive hybridization and NGS to generate species-specific DNA markers (Fig. 2). Suppressive subtractive hybridization was performed for two sets of selected plants. Each set included the authentic plant and its adulterant. The two sets of plants were *B. diffusa* with *T. portulacastrum* and *T. cordifolia* with *T. sinensis*, respectively. Briefly, the tester (authentic medicinal plant) and driver (adulterant species) DNA were quantified and subjected to suppressive subtractive hybridization and NGS library preparation using Ion Xpress™ Plus gDNA Fragment Library kit according to the manufacturer's protocol with minor modifications. 1 µg of DNA from the tester plant and 2 µg of DNA from the driver plant were fragmented using enzymatic digestion and purified. The tester DNA was adapter ligated and hybridized with driver DNA in the ratio of 1:20. Following hybridization, a double-stranded DNA-specific nuclease, namely dsDNase, was used to cleave hybridized fragments. Finally, using Platinum™ PCR SuperMix High Fidelity, the adapter ligated and subtracted tester DNA library was amplified. The amplified library was subjected to enrichment on Ion OneTouchTM ES System. The enriched sequence templates that have been amplified on Ion PI™ Ion Sphere™ Particles (ISPs) were sequenced on Ion 316™ Chip Kit v2 BC using the PGM platform.

### Data analysis

The sequencing data generated through the PGM machine were analyzed using SPAdes 3.12.0 (genome assembler). The assembled genome file was extracted in FASTQ format and was analyzed for quality using FastQC software. The FASTQ file was then subjected to quality assessment using the QUAST tool. The contigs with more than 500 bp were extracted and subject to BLAST analysis using the BLASTN program (Altschul et al. 1990). The unique contigs were selected and filtered based on the number of reads for *B. diffusa* and *T. cordifolia* and a mismatch percentage of less than 5%. Finally, the unique contigs obtained were subjected to primer design to generate DNA markers.

### Validation of DNA markers

The species-specific primers were designed for *B. diffusa* (18 sets of primers) and *T. cordifolia* (34 sets of primers) from unique species-specific sequences obtained from NGS data analysis. The *Boerhavia diffusa* L. primers were tested in 20 accessions of *B. diffusa* and *T. portulacastrum*. The PCR amplicons were subjected to validation by Sanger DNA sequencing on ABI 3130 sequencer. The sequence obtained from Sanger DNA sequencing was tested for similarity with the sequence obtained from NGS data analysis using BLAST tool analysis.

### The efficiency of DNA markers in the identification of plant species and detect adulterants

The primer sets developed for *B. diffusa* were tested for false negative and false positive amplification in 20 sets of *B. diffusa* and *T. portulacastrum* accessions collected from different geographical locations*.* Further, the developed primer sets were validated in five raw *B. diffusa* drug samples. The primer set designed for *T. cordifolia* was also tested for false negative and false positive amplification in 20 sets of *T. cordifolia* and *T. sinensis.* The developed primer sets were also validated in five raw drug samples of *T. cordifolia.*

## Results

### Sample collection and plant identification

The selected four plant species were collected from different regions of Southern India and given unique accession numbers (Tables S1 and S2). The herbarium of the selected plant species was stored at the Manipal School of Life Science, MAHE, Manipal, India. The germplasm was maintained in the greenhouse at Manipal School of Life Sciences, MAHE, Manipal, India.

### DNA barcoding

DNA barcoding of *B. diffusa, T. portulacastrum, T. cordifolia,* and *T. sinensis* was done using a universal DNA markers. The *nrITS* marker was used for all the four selected species, and the amplicon size was approximately 700 bp (Fig. S1). The *matK* marker was used for only *B. diffusa* and *T. cordifolia,* and the amplicon size was about 1000 bp, respectively (Fig. S2). Further, the DNA barcode sequences for *nrITS* and *matK* markers of *B. diffusa, T. portulacastrum, T. cordifolia,* and *T. sinensis* were submitted to the GenBank database, and the accession numbers are "OM639971", "OM639972", "MW362771", "OM639950", "MK947217", and "MN186382" (Table S3).

### Suppressive subtractive hybridization and NGS data analysis

**Fig. 3:**
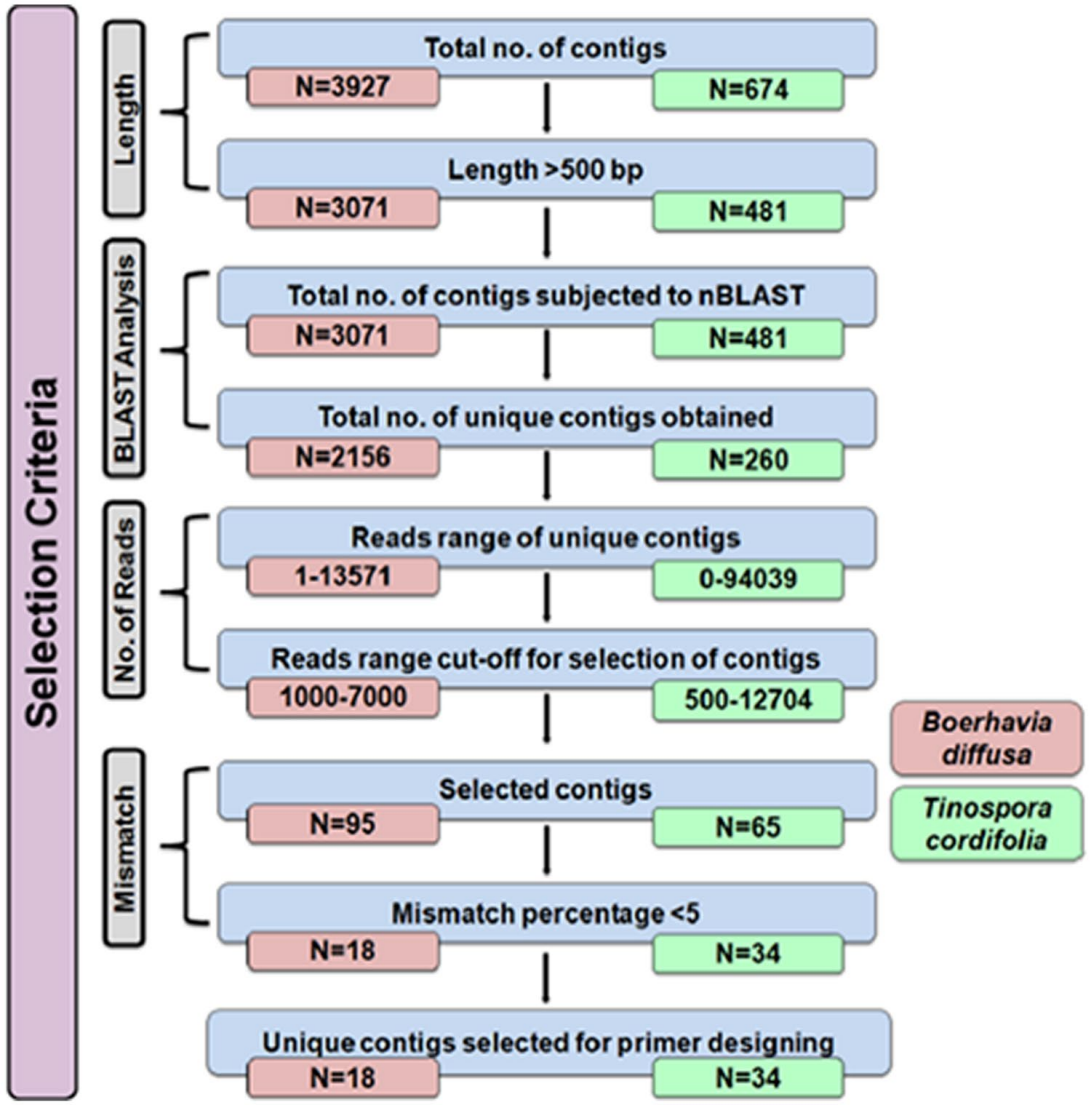
Criteria for selection of unique contigs from the NGS sequencing data

The plant materials from *B. diffusa* and *T. cordifolia* were used as a tester and *T. portulacastrum* and *T. sinensis* were used as a driver for the suppressive subtractive hybridization followed by NGS sequencing. The NGS sequencing yielded a data size of 444 Mb for *B. diffusa* and 53.3 Mb for *T. cordifolia.* The contig length, contig index, coverage, and GC content for subtracted genome are shown (Table S4). The contigs were generated using SPAdes 3.12.0 genome assembler, and the quality score was evaluated by FastQC software. We found 3071 contigs for *B. diffusa* and 481 for *T. cordifolia* after quality assessment by QUAST software. These contigs were subjected to a similarity search using the blastn tool, and 2156 unique contigs for *B. diffusa* and 260 unique contigs for *T. cordifolia* were identified. We obtained read counts ranging from 1 to 13,571 for *B. diffusa* and 0 to 94,039 for *T. cordifolia*. The contigs were filtered based on reads ranging from 1000 to 7000 for *B. diffusa* and 500 to 12,704 for *T. cordifolia*. Based on the read counts, we identified 95 unique contigs for *B. diffusa* and 65 unique contigs for *T. cordifolia. *Finally, these were filtered for a mismatch error rate of less than 5%, 18 unique contigs for *B. diffusa* and 34 unique contigs for *T. cordifolia* were selected for primer design (Fig. 3).

### Validation and efficiency of DNA markers

**Fig. 4:**
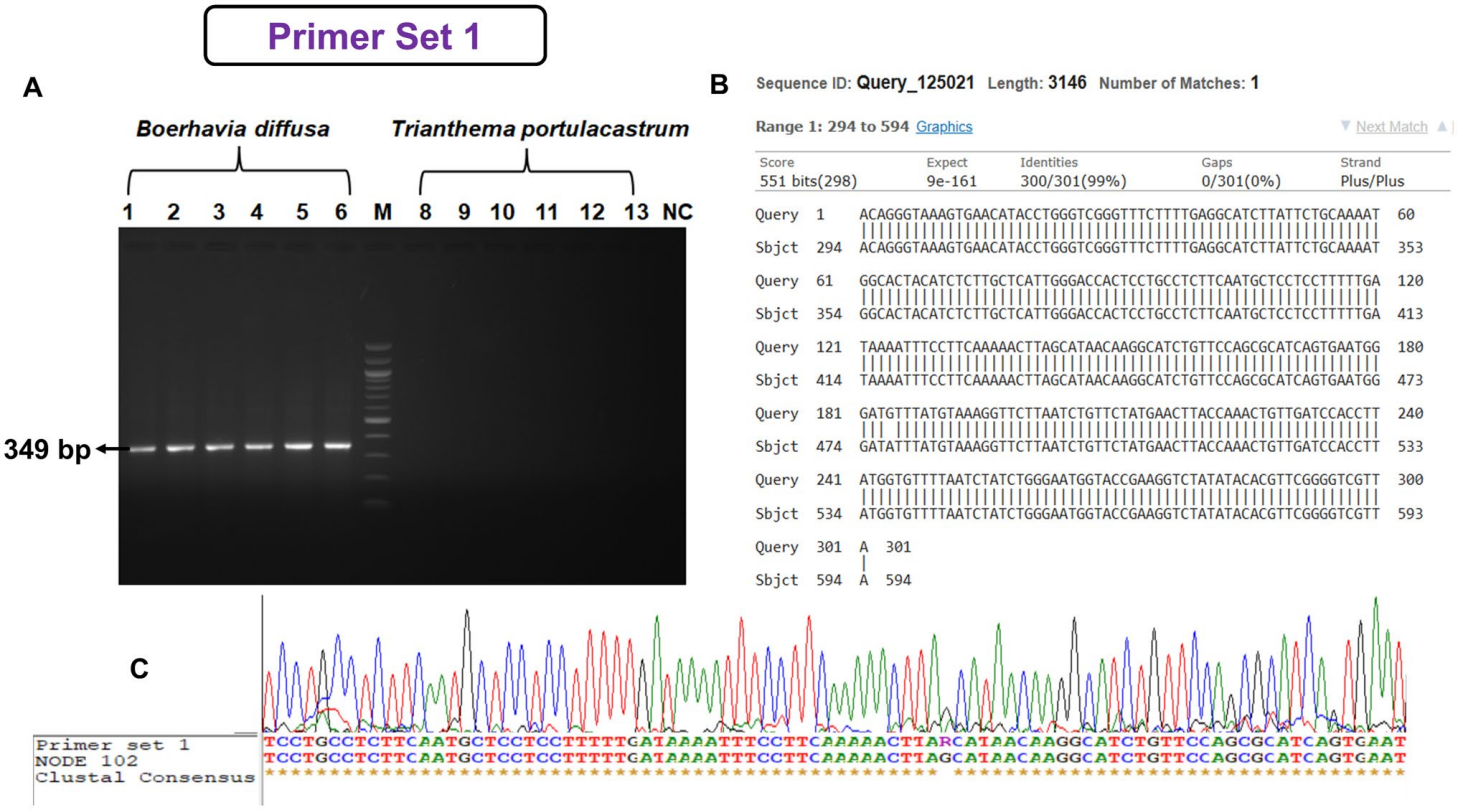
Validation of DNA marker of *Boerhavia diffusa* L. **A** Lanes 1–6: *B. diffusa* samples, M: 100 bp ladder, 8–13: *T. portulacastrum* samples, *NC* negative control, **B** BLAST alignment of *B. diffusa* sequences matched with the original contig sequence obtained by next-generation sequencing, **C** ClustalW alignment

**Fig. 5:**
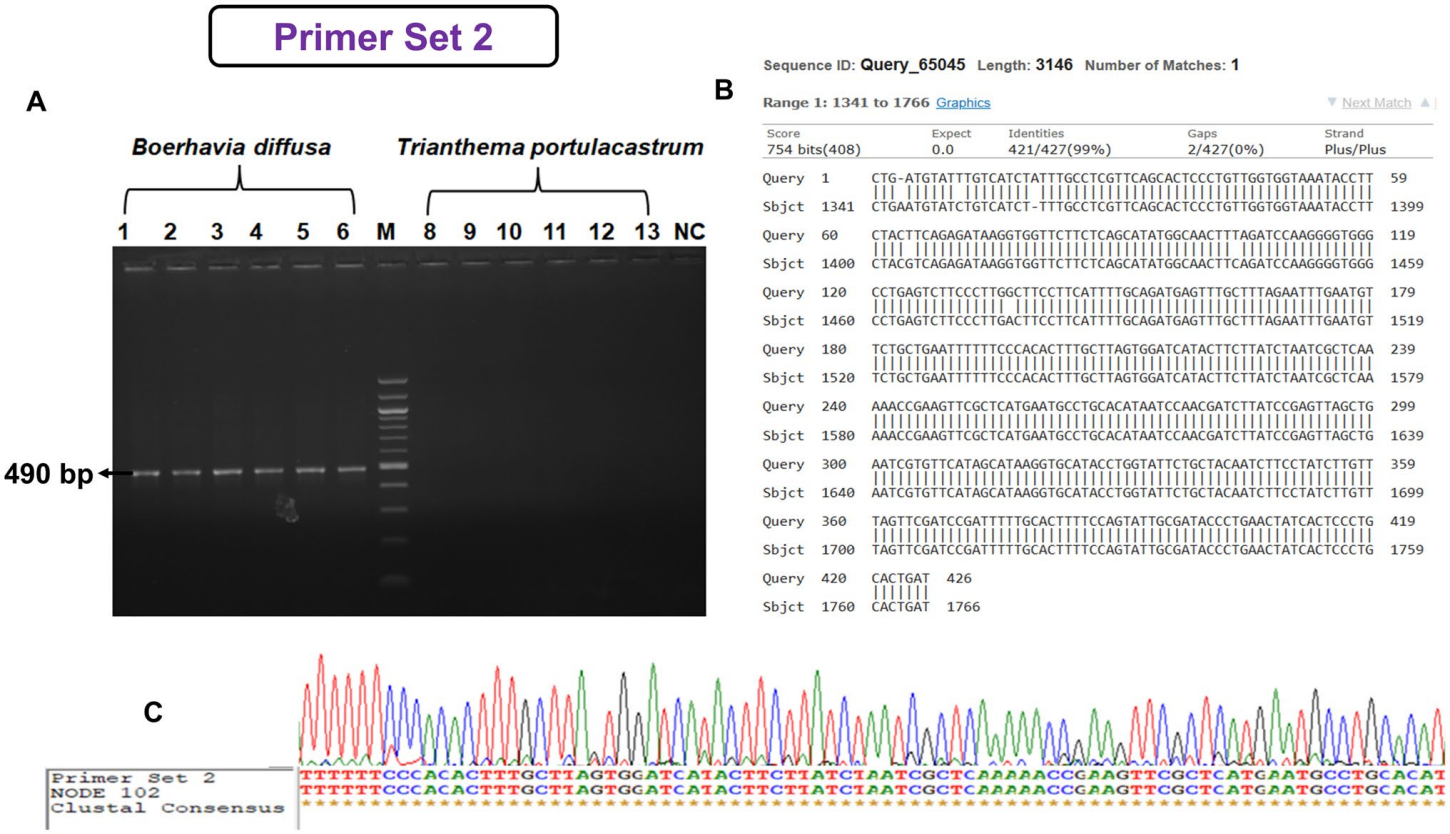
Validation of DNA marker of *Boerhavia diffusa* L. **A** Lanes 1–6: *B. diffusa* samples, M: 100 bp ladder, 8–13: *T. portulacastrum* samples, *NC* negative control, **B** BLAST alignment of *B. diffusa* L. sequences matched with the original contig sequence obtained by next-generation sequencing, **C** ClustalW alignment

**Fig. 6:**
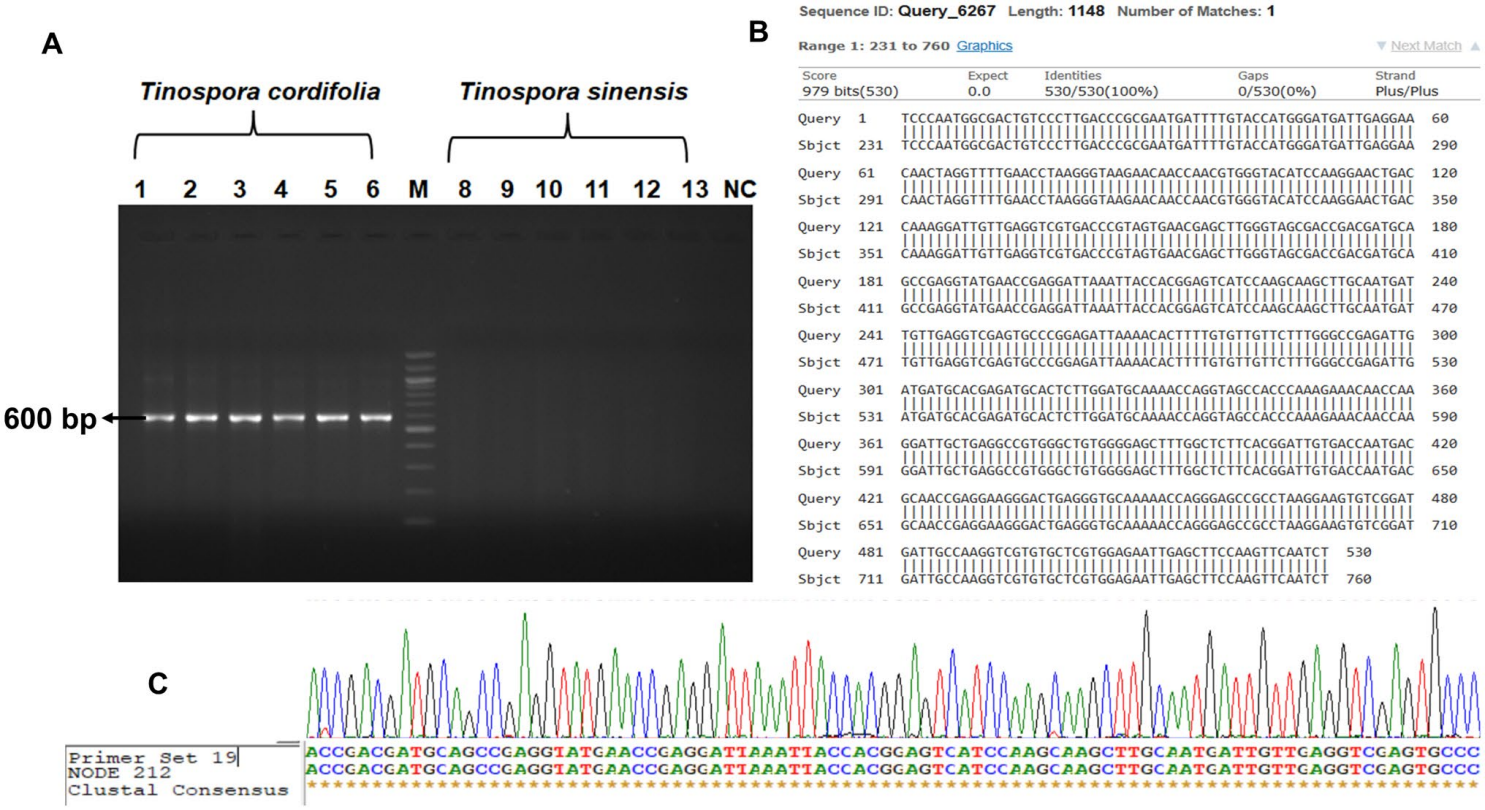
Validation of DNA marker of *Tinospora cordifolia* (Willd.) Miers. **A** Lanes 1–6: *T. cordifolia* samples, M: 100 bp ladder, 8–13: *T. sinensis* samples, *NC* negative control, **B** BLAST alignment of *T. cordifolia* sequences matched with the original contig sequence obtained by next-generation sequencing, **C** ClustalW alignment

Species-specific primers were designed for *B. diffusa* (18 sets of primers) and *T. cordifolia* (34 sets of primers) and were tested in 20 accessions of *B. diffusa* and *T. portulacastrum* and 20 accessions of *T. cordifolia* and *T. sinensis.* Out of 18 sets of primers for *B. diffusa,* we obtained 4 sets of primers that were specific to* B. diffusa*. However, out of four, only two showed 100% sensitivity and specificity (Table S5 and Table S6). Out of 20 sets of primers for *T. cordifolia* tested, we have obtained 1 species-specific primer set (Table S5 and Table S6). The results for the similarity of the sequences obtained from the specific markers and the sequence from the contigs were shown (Figs. 4, 5 and 6). The sequences obtained from the specific markers were deposited in the GenBank database under the accession numbers "OM728292", "OM728293", and "OM728294". The designed primer sets 1 and 2 for *B. diffusa* showed 100% sensitivity and 100% specificity for the 20 accessions of *B. diffusa*. Further, the developed primer sets were also validated in five raw drug samples of *B. diffusa*. The primer set designed for *T. cordifolia* was tested for false negative and false positive amplification in 20 accessions of *T. cordifolia* which showed 100% sensitivity and 100% specificity*.* The developed primer set was also validated in five raw drug samples of *T. cordifolia*.

## Discussion

Adulteration is a severe concern in the case of herbal medicines (Posadzki et al. 2013). Different methods like taxonomic identification, universal DNA barcodes, and metabolic fingerprinting have been used to avoid adulteration. However, the presence of the same vernacular names, similar morphology, and overlapping metabolites limits the universal application of these methods.

The present study developed a strategy to identify novel DNA-based markers with suppressive subtractive hybridization and a next-generation sequencing approach. The developed DNA marker was used to identify *B. diffusa* and *T. cordifolia* from its common adulterants *T. portulacastrum* and *T. sinensis*, respectively. Suppressive subtractive hybridization is commonly applied for the separation of DNA to distinguish between DNA samples of two closely related biological samples (Rebrikov et al. 2004). For instance, Li et al. 2004 used a suppressive subtractive hybridization method for screening species-specific DNA probes for species identification in five Dendrobium species. They effectively obtained different species-specific probes for each of the five species (Li et al. 2004). The method of suppressive subtractive hybridization has been extended to authenticate the population of GGL (Guilin, Guangxi) from the population of JSR (Shangrao, Jiangxi) by developing species-specific primers for *Dendrobium officinale* species (Ding et al. 2008). Ge et al. 2013 developed genome-specific molecular markers for tester *Lophopyrum elongatum* (Wheatgrass) and driver *Triticum aestivum* (Wheat) based on suppressive subtractive hybridization (Ge et al. 2013). In plants, suppressive subtractive hybridization coupled with microarray has been used to scan repetitive regions, tissue-specific cDNA, differentially regulated regions, etc. (Diatchenko et al. 1996; Sahebi et al. 2015).

Next-generation sequencing has allowed the massively parallel sequencing and analysis of various regions of the plant genome (Pareek et al. 2011). In the current investigation, we coupled the suppressive subtractive hybridization with next-generation sequencing to sequence the DNA fragments obtained by subtracting the adulterant plant's DNA from the tester plant's DNA. With bioinformatics tools, the de novo genome assembly was performed for the sequenced fragments. The fragments further yielded unique signatures for developing novel specific primers to identify the tester plants.

*Boerhavia diffusa* has many applications for managing inflammations, wounds, and expelling kidney stones, jaundice, and dyspepsia (Prachi et al. 2009; Ragi and Sahaya Shibu 2014). *B. diffusa* is commonly used in Punarnava churna in Ayurveda. The plant's roots can be an abortifacient, diuretic, laxative, analgesic, and anticonvulsant (Aslam 2017). On the other hand, *T. cordifolia* is used with the name Guduchi in Ayurveda and is found in higher altitudes (Rana et al. 2012). *T. cordifolia* harbors components like glycosides, aliphatic, diterpenoid lactones, steroids, and alkaloids in different parts like the stem, root, or sometimes whole plant (Upadhyay et al. 2010). This plant is of medicinal interest due to its anti-neoplastic, immunomodulatory, hepatoprotective, anti-malarial, anti-leprotic, anti-stress, anti-allergic, anti-oxidant, anti-arthritic, anti-inflammatory, anti-spasmodic, anti-periodic, and anti-diabetic activities (Saha and Ghosh 2012). Owing to these medicinal properties, there is a need to restrict or check the adulteration of *B. diffusa* and *T. cordifolia* with its common adulterant *T. portulacastrum* and *T. sinensis*, respectively.

## Conclusion

The novel DNA markers established from the current study are cost-effective, robust, and rapid and can discriminate the adulterant from the authentic plant species. It is a PCR-based identification technique and does not require sophisticated instruments like the Sanger DNA sequencer. 100% specificity and sensitivity of the developed markers highlight the strength of the established markers. Further, this strategy can be extrapolated to other plant species to generate unique DNA markers for authentication.

## Supplementary Information

Below is the link to the electronic supplementary material.

Supplementary file1 (PDF 224 KB)

## Data Availability

The sequence data for DNA markers that support the finding of this study have been deposited in "GenBank" with the accession numbers "OM728292", "OM728293", "OM728294" and the web links "https://www.ncbi.nlm.nih.gov.in/nuccore/OM728292", "https://www.ncbi.nlm.gov.in/nuccore/OM728293" and "https://www.ncbi.nlm.gov.in/nuccore/OM728294", respectively. The sequence data for universal DNA markers that support the finding of this study have been deposited in "GenBank" with the accession numbers "OM639971", "OM639972", "MW362771", "OM639950", "MK947217", "MN186382" and the web links "https://www.ncbi.nlm.gov.in/nuccore/OM639971", "https://www.ncbi.nlm.gov.in/nuccore/OM639972", "https://www.ncbi.nlm.gov.in/nuccore/MW362771", "https://www.ncbi.nlm.gov.in/nuccore/OM639950", "https://www.ncbi.nlm.gov.in/nuccore/MK947217", "https://www.ncbi.nlm.gov.in/nuccore/MN186382", respectively.
