# Supplementary material for: Development of DNA markers using next-generation sequencing approach for molecular authentication of Boerhavia diffusa L. and Tinospora cordifolia (Willd.) Miers
Source: 3 Biotech. 2023 Aug 15;13(9):304. doi: 10.1007/s13205-023-03732-7 (PMC10427588; doi:10.1007/s13205-023-03732-7)
Supplement: Supplementary file 1 — Supplementary file1 (PDF 224 KB) [file 13205_2023_3732_MOESM1_ESM.pdf]

Supplementary Information

Development of DNA Markers using Next Generation Sequencing Approach for Molecular Authentication of *Boerhavia diffusa* L. and *Tinospora cordifolia* (Willd.) Miers.

Table S1 Details of *Tinospora cordifolia* (Willd.) Miers., and *Tinospora sinensis* (Lour.) Merr. plant collection and storage

Sl.no.	Accession Number	Plant Name	Collection material	Stored/Grown
1	20170031A	*T. cordifolia*	Stem	Grown
2	20170037A	*T. cordifolia*	Stem	Grown
3	20180095A	*T. cordifolia*	Stem	Grown
4	20180109A	*T. cordifolia*	Stem	Grown
5	20180123A	*T. cordifolia*	Stem	Grown
6	20180132A	*T. cordifolia*	Stem	Grown
7	20190001A	*T. cordifolia*	Stem	Grown
8	20190034A	*T. cordifolia*	Stem	Grown
9	20190036A	*T. cordifolia*	Stem	Grown
10	20190069A	*T. cordifolia*	Stem	Grown
11	20190070A	*T. cordifolia*	Stem	Grown
12	20190074A	*T. cordifolia*	Stem	Grown
13	20190086A	*T. cordifolia*	Stem	Grown
14	20190087A	*T. cordifolia*	Stem	Grown
15	20190059A	*T. cordifolia*	Stem	Grown
16	20190067A	*T. cordifolia*	Stem	Grown
17	20190067B	*T. cordifolia*	Stem	Grown
18	20190083A	*T. cordifolia*	Stem	Grown
19	20190084A	*T. cordifolia*	Stem	Grown
20	20190085A	*T. cordifolia*	Stem	Grown
**Sl.no.**	**Accession Number**	**Plant Name**	**Collection material**	**Stored/Grown**
21	20180069A	*T. sinensis*	Stem	Grown
22	20180085A	*T. sinensis*	Stem	Grown
23	20180111A	*T. sinensis*	Stem	Grown
24	20190134A	*T. sinensis*	Stem	Grown
25	20190145A	*T. sinensis*	Stem	Grown
26	20190148A	*T. sinensis*	Stem	Grown
27	20190154A	*T. sinensis*	Stem	Grown
28	20190155A	*T. sinensis*	Stem	Grown
29	20190157A	*T. sinensis*	Stem	Grown
30	20190005A	*T. sinensis*	Stem	Grown
31	20190037A	*T. sinensis*	Stem	Grown
32	20190040A	*T. sinensis*	Stem	Grown

33	20190076A	*T. sinensis*	Stem	Grown
34	20190092A	*T. sinensis*	Stem	Grown
35	20190054A	*T. sinensis*	Stem	Grown
36	20190088A	*T. sinensis*	Stem	Grown
37	20190060A	*T. sinensis*	Stem	Grown
38	20190060B	*T. sinensis*	Stem	Grown
39	20190060C	*T. sinensis*	Stem	Grown
40	20190068A	*T. sinensis*	Stem	Grown

Table S2 Details of *Boerhavia diffusa* L., and *Trianthema portulacastrum* L., plant collection and storage

Sl.no.	Accession Number	Plant Name	Collection material	Stored/Grown
1	20170034A	*B. diffusa*	Whole plant	Grown
2	20190133A	*B. diffusa*	Whole plant	Grown
3	20170034B	*B. diffusa*	Whole plant	Grown
4	20170034C	*B. diffusa*	Whole plant	Grown
5	20170034D	*B. diffusa*	Whole plant	Grown
6	20170034E	*B. diffusa*	Whole plant	Grown
7	20180063A	*B. diffusa*	Whole plant	Grown
8	20180064A	*B. diffusa*	Whole plant	Grown
9	20180065A	*B. diffusa*	Whole plant	Grown
10	20180067A	*B. diffusa*	Whole plant	Grown
11	20180068A	*B. diffusa*	Whole plant	Grown
12	20180083A	*B. diffusa*	Whole plant	Grown
13	20190140A	*B. diffusa*	Whole plant	Grown
14	20180089A	*B. diffusa*	Whole plant	Grown
15	20180090A	*B. diffusa*	Whole plant	Grown
16	20180094A	*B. diffusa*	Whole plant	Grown
17	20180105A	*B. diffusa*	Whole plant	Grown
18	20180105B	*B. diffusa*	Whole plant	Grown
19	20190135A	*B. diffusa*	Whole plant	Grown
20	20190135B	*B. diffusa*	Whole plant	Grown
**Sl.no.**	**Accession Number**	***Plant Name***	**Collection material**	**Stored/Grown**
21	20180070A	*T. portulacastrum*	Whole plant	Grown
22	20180071A	*T. portulacastrum*	Whole plant	Grown
23	20180073A	*T. portulacastrum*	Whole plant	Grown
24	20180073B	*T. portulacastrum*	Whole plant	Grown
25	20180073C	*T. portulacastrum*	Whole plant	Grown
26	20180074A	*T. portulacastrum*	Whole plant	Grown
27	20180074B	*T. portulacastrum*	Whole plant	Grown
28	20180077C	*T. portulacastrum*	Whole plant	Grown
29	20180121A	*T. portulacastrum*	Whole plant	Grown
30	20180122A	*T. portulacastrum*	Whole plant	Grown
31	20180128A	*T. portulacastrum*	Whole plant	Grown
32	20180128B	*T. portulacastrum*	Whole plant	Grown

33	20190142A	*T. portulacastrum*	Whole plant	Grown
34	20190142B	*T. portulacastrum*	Whole plant	Grown
35	20190142C	*T. portulacastrum*	Whole plant	Grown
36	20190143B	*T. portulacastrum*	Whole plant	Grown
37	20190146A	*T. portulacastrum*	Whole plant	Grown
38	20190146B	*T. portulacastrum*	Whole plant	Grown
39	20190146C	*T. portulacastrum*	Whole plant	Grown
40	20190146D	*T. portulacastrum*	Whole plant	Grown

Table S3 Details of DNA barcoding of selected plant species using universal DNA markers

Species	Tissue	GenBank Accession numbers	
		***nrITS* marker**	***matK* marker**
Boerhavia diffusa L.	Whole plant	OM639971	MK947217
Trianthema portulacastrum L.	Whole plant	OM639972	-
Tinospora cordifolia (Willd.) Miers	Stem	MW362771	MN186382
Tinospora sinensis (Lour.) Merr.	Stem	OM639950	-

Table S4 The quality assessment of the subtracted genome using QUAST software.

Attributes	*Boerhavia diffusa* L.	*Tinospora cordifolia* (Thunb.) Miers
Contigs selected	3071	481
Contigs (>= 0 bp)	3927	674
Contigs (>= 1000 bp)	1177	254
Contigs (>= 5000 bp)	47	16
Contigs (>= 10000 bp)	15	2
Contigs (>= 25000 bp)	3	1
Contigs (>= 50000 bp)	0	0
Largest contig	40694	28243
Total length	3786275	768143
Total length (>= 0 bp)	3993383	822035
Total length (>= 1000 bp)	2237538	578998
Total length (>= 5000 bp)	504110	136388
Total length (>= 10000 bp)	291763	39709
Total length (>= 25000 bp)	111823	28243
Total length (>= 50000 bp)	0	0
N50	1115	1798
N75	848	1006
L50	850	105
L75	1838	252
GC (%)	37.76	39.32

Table S5 The primer details of the selected DNA markers

Plant Name	Primer Name	Primer	Sequence (5'-3')	Primer Length (bp)	Amplicon size (bp)	GenBank Accession number
Boerhavia diffusa L.	Primer Set 1	Forward	TGACCCAAGGTCAATGAGTG	20	349	OM728292
		Reverse	TAACGACCCCGAACGTGTAT	20		
	Primer Set 2	Forward	TTTCCTGAGCCGATGTCTTT	20	490	OM728293
		Reverse	TCTGATCAGTGCAGGGAGTG	20		
Tinospora cordifolia (Willd.) Miers	Primer Set	Forward	TGCGTATCGTGGTGTATCGT	20	600	OM728294
		Reverse	CAGCCCACGAAGATTGAACT	20		

Table S6 Sensitivity and specificity of selected DNA markers

Plant Name	Primer Name	Sensitivity (%)	Specificity (%)
Boerhavia diffusa L.	Primer Set 1	100	100
	Primer Set 2	100	100
Tinospora cordifolia (Willd.) Miers	Primer Set	100	100

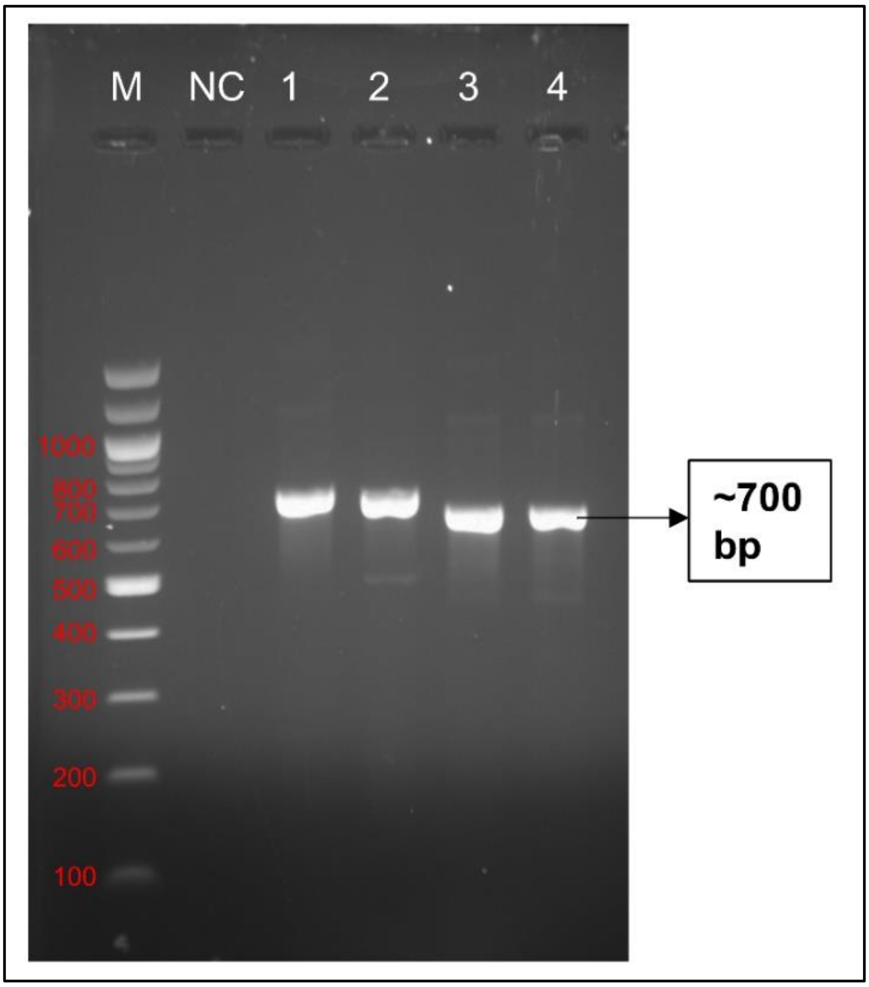

Fig. S1 PCR for the *nrITS* region of DNA samples. Lane 1. M-100 bp ladder, Lane 2. NC-Negative control, Lane 3. 1-*Boerhavia diffusa*, Lane 4. 2-*Trianthema portulacastrum*, Lane 5. 3-*Tinospora cordifolia*, Lane 6. 4-*Tinospora sinensis*

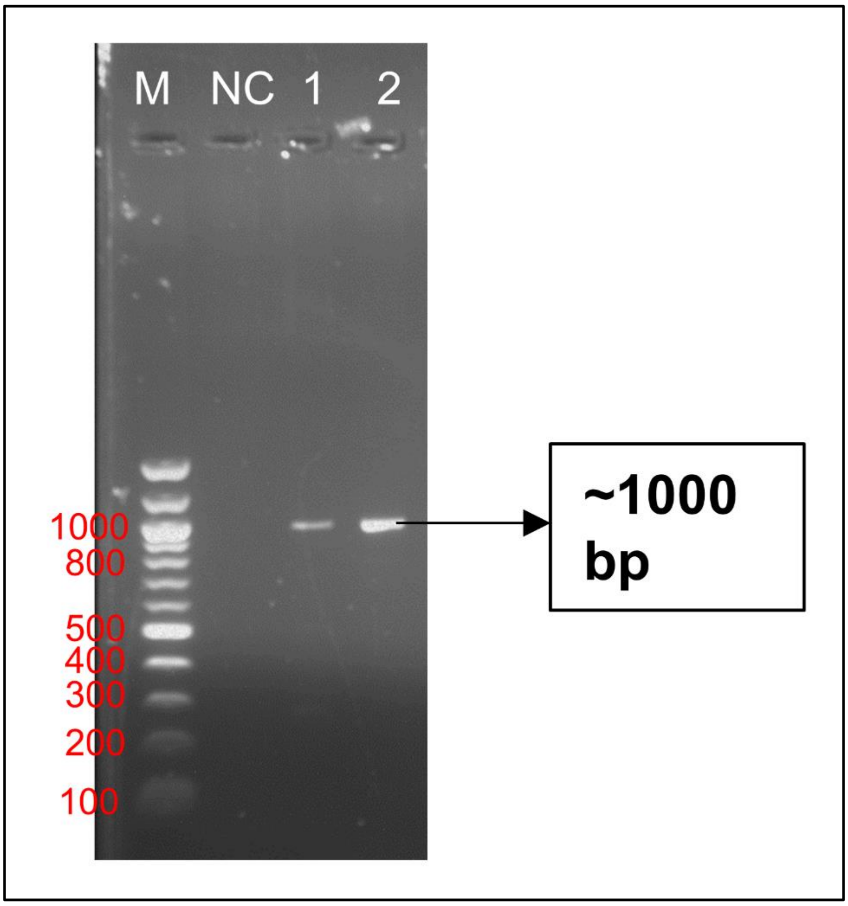

Fig. S2 PCR for the *matK* region of DNA samples. Lane 1. M-100 bp ladder, Lane 2. NC-Negative control, Lane 3. 1-*Boerhavia diffusa*, Lane 4. 2-*Tinospora cordifolia*
